# Correction: Microtubules and Gαo-signaling modulate the preferential secretion of young insulin secretory granules in islet β cells via independent pathways

**DOI:** 10.1371/journal.pone.0314639

**Published:** 2025-02-06

**Authors:** Ruiying Hu, Xiaodong Zhu, Mingyang Yuan, Kung-Hsien Ho, Irina Kaverina, Guoqiang Gu

The quantification data in the S1 Table are incorrect. Please view the correct S1 Table below.

## Supporting information

S1 Table (XLSX)
