# Supplementary figures and images for: Microtubules and Gαo-signaling modulate the preferential secretion of young insulin secretory granules in islet β cells via independent pathways
Source: PLoS One. 2021 Jul 22;16(7):e0241939. doi: 10.1371/journal.pone.0241939 (PMC8297875; doi:10.1371/journal.pone.0241939)

Raw images of Figure 4E.

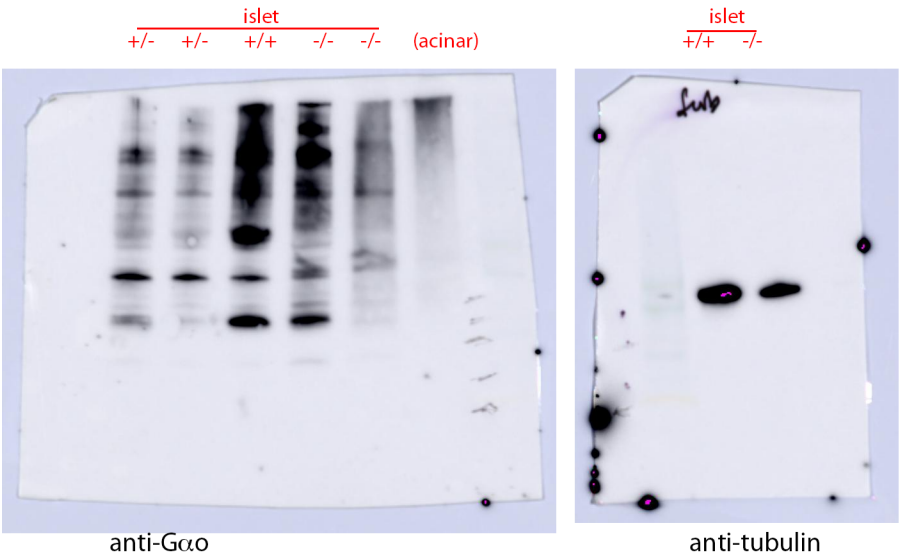

Supplement: S1 Fig — This Fig shows the western-blot assay of Gαo protein in control and Gαo mutant islets. A portion of this images was used in Fig 4E. (PDF) [file pone.0241939.s001.pdf]
