# Supplementary material for: Microtubules and Gαo-signaling modulate the preferential secretion of young insulin secretory granules in islet β cells via independent pathways
Source: PLoS One. 2021 Jul 22;16(7):e0241939. doi: 10.1371/journal.pone.0241939 (PMC8297875; doi:10.1371/journal.pone.0241939)
Supplement: Attachment — Submitted filename: Gu et al, 2020.docx [file pone.0241939.s003.docx]

To the Authors

- The introduction is written well and puts the study appropriately in the context of the existing literature. At line 49 the authors state that beta cells have 10000 insulin granules citing Olofsson et al. - since there are other studies out there showing somewhat divergent numbers it would be appropriate to cite also these studies.

- In the results the authors show that nocodazole treatment leads to the expected disruption of microtubule filaments. When additionally treated with high glucose, insulin granules are only visible in the periphery ('docked') of the cells. This is shown by IF stainings and EM of islets (fig. 1 and 2). To measure SG density the drawing of lines is not ideal. The authors should surround the cells and calculate the SG density in the full area excluding the nucleus. Also, In the material and methods section, the authors state that (line 438-439) the 'lines were drawn to avoid the nuclei,..' however the cells in figure 1C seem to do exactly that. The authors should clarify this.

- The authors state at line 149 to having observed 'significant reduction in total IG levels...' as seen in figure 1D. To make a proper statement, the authors should additionally show the integrated values of their measurement, or ideally integrate the signal of the entire cell, since signal along the line may be heavily biased by where the line is set. The figure legend of figure 1 should state if the SD or the SEM is shown.

- Figure 2 replicates the data from figure 1 using electron micrographs. The authors state that the lack of microtubules following 12 h treatment with nocodazole (NOC) leads to a relative increase of IGs at the PM and respective reduction in the center of the cells. This effect is attributed to 'slow-but-detectable random IG movement' (line 155-156). First of all,

a major question is whether after 12 h nocodazole treatment the cells still secrete insulin. This long treatment may lead to also cause fragmentation of the Golgi and block insulin synthesis and IG biogenesis. This is especially relevant since the authors state before that there is a reduction of total insulin. Notably, in figure 2H the ER looks very stressed.

As a NOC treatment for 12 h seems to us very long, the authors should test whether granules and/or insulin are still properly generated under these conditions, or if the relative increase of PM IGs is the result of blocked biogenesis. In addition, MT depletion could also block the retrograde transport of IGs destined to degradation toward the perinuclear region, where endolysosomes are enriched. These two effects combined may account for IG depletion in the perinuclear region without confuting the role of MTs in promoting the centrifugal transport of newly generated IGs toward the PM.

- The authors then investigate the secretion of old IGs under conditions of blocked protein synthesis and/or lack of microtubules. To this aim beta cells were treated for 3 h with CHX and an additional hour with NOC. It is not clear why in figures 1&2 the NOC treatment was done for 12 h and here 1 h is sufficient to depolymerize MTs (figure 3). The authors should state the reasoning for this discrepancy in the protocols. The authors then measure the secreted insulin, assuming that 'newly synthesized insulin' should be secreted within 2-4 h. As expected, less insulin is secreted when protein biogenesis is blocked with CHX. Notably additional treatment with NOC further increased insulin secretion. The number of replicates is low and, therefore, the individual experiments should be shown with a dot plot (or similar; with SD rather than SEM). Non-parametric testing may be more suitable with low number of replicates.

- The authors go on to directly measure the secretion of old and new IGs. To this aim, islets were labelled overnight (12 h) with 3H-leucine, then chased for 2 h and then used for GSIS (about 2 h). Figure 3F shows the 'relative amount of radioactive insulin...' (line 241-242) to be lower for NOC vs DMSO samples. It is not readily clear from the text if these data are representing the medium or the islets and this should be better explained for clarification. Also here the individual values should be shown as bar graphs with SEM may not be representative.

- At line 254-256 the authors state that the 12 h window prior to the GSIS assay is considered to be new IGs. In this case, how can the authors make a statement about old IGs, if all the labelled IGs (see protocol above) should fall in the 12 h window of 'new IGs'? In other words, if 12 h old IGs are considered to be newly-synthesized, the authors would not detect old IGs using this approach. The authors should clarify this point.

- Next, the authors investigate a potential link between the MT-network and the G protein subunit Gao as the latter may regulate MTs. To address this, the authors replicate the data from figure 3 using a mutated Gao mouse line. They find increased secretion of 'old IGs' (similar to figure 3, the nomenclature needs to be clarified and data points shown in another format than bar graphs with SEM due to the low replication number) in isolated islets of the mutant mice upon high glucose. In figure 4F, the authors test the secretion of insulin in mutant vs control islets with or without NOC. At low glucose, the authors should also consistently add a statistical test, as it appears that NOC here reduces the secretion. With high glucose and potassium treatment, the insulin secretion is expectedly increased and is even further increased when NOC is included. However, the authors should test if there is a difference in secretion already between islets of mutant and control mice, to avoid falsely attributing the secretion difference between mutant and control islets to NOC, when there may already be an underlying effect in the mutation itself.

- Furthermore, the authors investigate a potential effect of mutant Gao on the MT network. Measuring MT density and stability, the authors could not detect any difference between Gao mutant and control islets.

- Finally, the authors show that NOC treatment induces a relative increase of PM IGs compared to IGs in the cell body. Using radiolabelling, the authors demonstrate that 12 h old IGs are increasingly released upon glucose stimulation and/or NOC treatment. Although Gao mutant islets appear to increase insulin secretion (total or 12 h old IGs), the effects appear to be additive, rather than related to the MT-mediated effects of NOC.

In general, data representation must be improved, for example using dot plots rather than bar graphs, especially where only low replicates are available. In these situations, non-parametric testing is superior to parametric testing (for example Mann-Whitney vs t-test).

The final paragraphs should speculate a bit more about the role of Gao to put it into context here, as the final result suggests no connection to MTs.

Minor

- It would be preferable to replace the term “IGs” to “insulin SGs”, since the latter more commonly used.

- the term "docked" SGs should be avoided whenever the analyzed images are obtained from  fixed samples. In this instance one cannot indeed determine the residence time at the PM of the organelles, which may just be transient visitors. Moreover, “docking” implies a proximity <20 nm, i.e. below the resolution of fluorescence microscopy.
