# Supplementary material for: Microtubules and Gαo-signaling modulate the preferential secretion of young insulin secretory granules in islet β cells via independent pathways
Source: PLoS One. 2021 Jul 22;16(7):e0241939. doi: 10.1371/journal.pone.0241939 (PMC8297875; doi:10.1371/journal.pone.0241939)
Supplement: Attachment — Submitted filename: Galphao-PLOSone_rebuttal.docx [file pone.0241939.s004.docx]

**Overall responses:**

We thank the editor for the opportunity of revising this manuscript. We also thank the reviewers for their constructive suggestions to improve this paper. In this revised version, all the questions raised by the reviewers were addressed one-by-one with new data and/or textual revision.

***Reviewer #1:***

*This paper dealing with insulin granule dynamics is a follow-up study from Du’s group of a recent paper on microtubule (ref 34) and an older study on Galphao (ref 15) and as such part the data partially recapitulate previous observations. The authors show here by using nocodazole treatment of mouse pancreatic islets or islets recovered from Galphao mouse that the intracellular distribution of insulin granules is modified. Following these observations, they further blocked protein synthesis with cycloheximide and conclude that older insulin granules are preferentially secreted when microtubules are disrupted. Although conceptually interesting the paper suffers from several weaknesses among which overstatement and overinterpretation of the results is a major issue. Indeed, in my opinion the methods used do allow to convincingly support the interpretation made on insulin granule age differences.*

**Response:** We thank the reviewer for pointing out both the novelty and issues in the manuscript. In this revised version, we have added more data and revised the text to address these concerns. For easier following, we have numbered the critiques from this reviewer according to the order of their appearance.

***Critique 1:*** *The description of the image analysis for Figure 1 fails to describe how the confocal plan was selected for each cell as this could largely affect the IG density whether the image is captured in the middle part of the cells or close to either top or bottom parts.*

**Response:** In order to avoid making subjective selection of focal plans, we have now replaced the original line-scan assay with a new method (based on the suggestion by reviewer #2 – see his/her point #2 below). Briefly, we took single slice confocal images to visualize the plasma membranes (stained for Ecadherin) and insulin in beta cells. We then measured the portion of insulin (IF signals) that localized within a zone that is close to the plasma membrane (±1 micrometer) in all beta cells within a specific field. This new method avoids the complication caused by the presence of nuclei or artificial focal plan selection. Please see revised Figure 1 for details.

***Critique 2:*** *…Furthermore, the authors mention in the method section that the nuclear areas are avoided, but how are those areas defined is not mentioned. No nuclear staining seems to have been performed. Finally, it is not possible to identify the condition for which the values are statistically different in panel D/E.*

**Response:** We apologize for not including the DAPI (nuclear) signals in the original version. New images with DAPI signals were presented in the revised figure. To address the rest of the issues in quantification, we have adopted a new quantification method for pair-wise comparison under different conditions (see above and revised Figure 1).

***Critique 3:*** *The treatment of CHX is problematic as the authors did not should quantify its effect on novel granule biogenesis. Further the authors of ref 63 mention that this treatment actually specifically affects a short half-life protein important to ensure optimal exocytosis, suggesting that the present observation may not be specific to newly generated granules.*

**Response:** We have now included the data showing decreased radioactive amino acids incorporation into islet proteins (revised Figure 3F), which include insulin and other proteins.

For the same reason, we agree with the non-specificity of CHX treatment. Thus, our experimental design was to compare GSIS under (CHX + NOC) and (CHX + DMSO), with CHX inhibits protein synthesis while NOC disrupt MTs. The finding is that the presence of CHX did not eliminate the NOC-potentiated GSIS, which is consistent with (but not prove) the conclusion that older vesicles have increased chance to be secreted in the absence of MT. This point was further corroborated by the radio-labeling studies in original Figure 3F. We cannot exclude the possibility that the lack of MTs may have unpredictable effect(s) on the half-life/function of proteins that regulate secretion of vesicles of different age. But this possibility can be broadly interpreted as MTs impacting older vesicle secretion indirectly. These points were made clear in the revised text.

***Critiques 4:*** *According to this model why is there more newly synthesized granules released in the low glucose condition?*

**Response:** We assume that the reviewer meant why beta cells release a relatively higher portion (but not absolute number) of newly synthesized SGs under low glucose. We do not know the exact mechanism. But this observation is consistent with our and others’ models. E.g., some data have suggested the heterogeneity of insulin SGs (eg, those of Solimena and ours). Newly synthesized vesicles are more mobile, providing higher probability to localize to the periphery of beta cells for release ([10.1073/pnas.1409542112](https://doi.org/10.1073/pnas.1409542112)). In addition, a fraction of newly synthesized vesicles may be more sensitive to Ca2+-induced secretion and can be preferentially released under low glucose ([10.1016/j.devcel.2018.03.013](https://dx.doi.org/10.1016%2Fj.devcel.2018.03.013)). These points were made clear in the revised manuscript.

***Critique 5:*** *Glucose is missing in Figure 3D/E to indicate what the concentrations stand for!*

**Response:** These details are added (10 mM glucose for islet incubation) and 2.8 mM (G2.8) or 20 mM (G20) glucose incubation during secretion assays.

***Critique 6:*** *The expression level of Galphao and the remaining peptide should be shown by western blot.*

**Response:** The western results were shown in revised Figure 4E.

***Critique 7:*** *As such with the limited data of Figure 4, the conclusion on line 287-289 “Thus, different pools of IGs were mobilized/secreted in response to Galphao inactivation and MT disassembly, implying that Galphao does not directly regulate MTs in betta cells” appears to be an overstatement at this time of the study as it is also possible that Galphao acts through a moderate MT depolymerization that is boosted by nocodazole treatment.*

**Response:** We thank the reviewer for highlighting this overstatement. We have changed the text to “These data are consistent with a model that Galphao inactivation and MT-disassembly facilitate the release a different sub-pools of insulin vesicles. Otherwise, we would have found that the dual manipulations would not have further enhanced GSIS than MT-treatment or Galphao inactivation separately. To corroborate this conclusion, we next examined the interaction between Galphao activation and MT stability.”

***Critique 8:*** *Indeed, although not statistically significant there seem to be tendency that Galphao affected microtubule density and stability. Once more the data do not fully support the author’s conclusion.*

**Response:** We thank the reviewer for pointing out trends of higher MT density (p=0.31) and stability (p=0.24) of MTs in the absence of Galphao signaling. These imply a weak role of Galphao in stabilizing MTs. Yet Galphao inactivation increases, while MT stabilization inhibits, GSIS. Thus, although we agree that the data in original Figure 5 cannot exclude the effect of Galphao on MT, the overall findings are consistent with a model that Galphao does not inhibit GSIS by stabilizing the MTs. We have made these points clear in the revised manuscript.

***Critique 9:*** *Finally, another point that is at least worth discussing is the observation that Galphao was found associated with secretory granules membranes from different cell types including chromaffin cells. In these cells, it was shown that Galphao negatively regulates granule secretion by modulating cortical actin polymerization. The authors should discuss these findings and comment on the distribution of Galphao in pancreatic beta cells.*

**Response:** We have expanded the discussion to include the potential play of cortical actin/Galphao distribution.

**Reviewer #2:**

**Overall response:** We thank the reviewer for the constructive comments and suggestions. The raised questions are addressed as below.

***Critique 1:*** *- The introduction is written well and puts the study appropriately in the context of the existing literature. At line 49 the authors state that beta cells have 10000 insulin granules citing Olofsson et al. - since there are other studies out there showing somewhat divergent numbers it would be appropriate to cite also these studies.*

**Response:** Several other references are now added (showing the presence of 5,000-13,000 granules/normal beta cell).

***Critique 2****- In the results the authors show that nocodazole treatment leads to the expected disruption of microtubule filaments. When additionally treated with high glucose, insulin granules are only visible in the periphery ('docked') of the cells. This is shown by IF stainings and EM of islets (fig. 1 and 2). To measure SG density the drawing of lines is not ideal. The authors should surround the cells and calculate the SG density in the full area excluding the nucleus. Also, In the material and methods section, the authors state that (line 438-439) the 'lines were drawn to avoid the nuclei,..' however the cells in figure 1C seem to do exactly that. The authors should clarify this.*

**Response:** We have now quantified insulin distribution – following this suggestion – as portion of insulin that locate close to the plasma membrane (over the total, also see answer to reviewer #1, point 1). This approach avoids not only the issues of line-scan, but also the complicating issue of measuring insulin levels in nuclear areas. Note that in the original Figure 1C, we unwisely presented Z-stack images. These have now been replaced with single optical slices.

***Critique 3-*** *The authors state at line 149 to having observed 'significant reduction in total IG levels...' as seen in figure 1D. To make a proper statement, the authors should additionally show the integrated values of their measurement, or ideally integrate the signal of the entire cell, since signal along the line may be heavily biased by where the line is set. The figure legend of figure 1 should state if the SD or the SEM is shown.*

**Response:** A new panel showing the relative insulin IF signals was now included (Figure 1E-G). For quantification, SEMs were used here and noted in figure legend (1G, H).

***Critique 4****- Figure 2 replicates the data from figure 1 using electron micrographs. The authors state that the lack of microtubules following 12 h treatment with nocodazole (NOC) leads to a relative increase of IGs at the PM and respective reduction in the center of the cells. This effect is attributed to 'slow-but-detectable random IG movement' (line 155-156).* ***(a)*** *First of all, a major question is whether after 12 h nocodazole treatment the cells still secrete insulin.* ***(b)*** *This long treatment may lead to also cause fragmentation of the Golgi and block insulin synthesis and IG biogenesis. This is especially relevant since the authors state before that there is a reduction of total insulin. Notably, in figure 2H the ER looks very stressed. As a NOC treatment for 12 h seems to us very long, the authors should test whether granules and/or insulin are still properly generated under these conditions, or if the relative increase of PM IGs is the result of blocked biogenesis.* ***(C)*** *In addition, MT depletion could also block the retrograde transport of IGs destined to degradation toward the perinuclear region, where endolysosomes are enriched. These two effects combined may account for IG depletion in the perinuclear region without confuting the role of MTs in promoting the centrifugal transport of newly generated IGs toward the PM.*

**Response:**

(a) We have added the GSIS data (Figure 1B), showing that beta cells after 12-hour NOC treatment (with 20 mM glucose) secrete bigger portion of insulin than controls.

(b) We apologize for our confusing conclusion. The reviewer is correct that NOC-treatment will lead to the fragmentation of Golgi and largely-reduced insulin biosynthesis (published in Trogden et al 2019). Thus, examining the relative insulin distribution alone (Figure 1C, D) cannot determine if bulk IG movement still occurs in the absence of MTs. This is the main reason why we introduced TEM to quantify the absolute number (not the portion) of IGs that localize very-close to the PM. The finding is that more IGs near PM were observed in the absence of MTs (Figure 2C, I), even though the latter conditions also had higher levels of secretion (Figure 1F). Thus, these combined results support a conclusion MTs reduces PM-localized IGs. These points are made clear in the revised text.

(C) We thank the reviewer for pointing out the possible roles of MTs in retrograde IG movement. This point is discussed in the revised text (bottom of page 9 and top of page 11).

***Critique 5*** - The authors then investigate the secretion of old IGs under conditions of blocked protein synthesis and/or lack of microtubules. To this aim beta cells were treated for 3 h with CHX and an additional hour with NOC. It is not clear why in figures 1&2 the NOC treatment was done for 12 h and here 1 h is sufficient to depolymerize MTs (figure 3). (a) The authors should state the reasoning for this discrepancy in the protocols. The authors then measure the secreted insulin, assuming that 'newly synthesized insulin' should be secreted within 2-4 h. As expected, less insulin is secreted when protein biogenesis is blocked with CHX. Notably additional treatment with NOC further increased insulin secretion. (b) The number of replicates is low and, therefore, the individual experiments should be shown with a dot plot (or similar; with SD rather than SEM). Non-parametric testing may be more suitable with low number of replicates.

Response:

(a) According to published data (Solimena, Rutter, ourown, etc), the portion of long-range moving insulin vesicles is very small, poising challenges for quantifying bulk vesicle localization changes. Thus, a long-period of time (12 hours) is expected to detect such changes – although 1-hour is sufficient to depolymerize MTs. For the CHX-inhibition assays, we tried to minimize the impact of reduced non-insulin biosynthesis (reported in ref 63) while also sufficiently reducing insulin synthesis for experimental detection, making us to choose 3-hours. This thought process is presented in the revised text.

(b) Data processing has been presented as suggested (all data with n<8 were presented as scattered plot, with average indicated).

***Critique 6*** - The authors go on to directly measure the secretion of old and new IGs. To this aim, islets were labelled overnight (12 h) with 3H-leucine, then chased for 2 h and then used for GSIS (about 2 h). Figure 3F shows the 'relative amount of radioactive insulin...' (line 241-242) to be lower for NOC vs DMSO samples. It is not readily clear from the text if these data are representing the medium or the islets and this should be better explained for clarification. Also here the individual values should be shown as bar graphs with SEM may not be representative.

**Response:** The presented data show the secreted insulin in GSIS assays after the chase. The data presentation is also changed as suggested. These are now clarified.

***Critique 7*** - At line 254-256 the authors state that the 12 h window prior to the GSIS assay is considered to be new IGs. In this case, how can the authors make a statement about old IGs, if all the labelled IGs (see protocol above) should fall in the 12 h window of 'new IGs'? In other words, if 12 h old IGs are considered to be newly-synthesized, the authors would not detect old IGs using this approach. The authors should clarify this point.

**Response:** We apologize for the confusion. We have revised the term as young – vs. old vesicles. Note that we have also replaced the data with new assays that label SGs within a 4-hour (instead of 12) labeling window (revised figure 3G and 4F). This new scheme allows us to more clearly define young vs. old SGs.

***Critique 8*** - Next, the authors investigate a potential link between the MT-network and the G protein subunit Gao as the latter may regulate MTs. To address this, the authors replicate the data from figure 3 using a mutated Gao mouse line. They find increased secretion of 'old IGs' (similar to figure 3, the nomenclature needs to be clarified and data points shown in another format than bar graphs with SEM due to the low replication number) in isolated islets of the mutant mice upon high glucose. In figure 4F, the authors test the secretion of insulin in mutant vs control islets with or without NOC. At low glucose, the authors should also consistently add a statistical test, as it appears that NOC here reduces the secretion.

**Response:** We have clarified the namenclature and data presentation format is changed as suggested. The low-glucose data is analyzed by new statistical analysis as well (note that the presented data are relative radioactivity/amount of insulin, but not absolute secretion).

***Critique 9*** - With high glucose and potassium treatment, the insulin secretion is expectedly increased and is even further increased when NOC is included. However, the authors should test if there is a difference in secretion already between islets of mutant and control mice, to avoid falsely attributing the secretion difference between mutant and control islets to NOC, when there may already be an underlying effect in the mutation itself.

**Response:** The Galphao mutant islets does secrete more insulin than controls (previously published and repeated here again). The statistical analysis is now added in the revised figure 3F.

***Critique 10*** *- Furthermore, the authors investigate a potential effect of mutant Gao on the MT network. Measuring MT density and stability, the authors could not detect any difference between Gao mutant and control islets.*

*- Finally, the authors show that NOC treatment induces a relative increase of PM IGs compared to IGs in the cell body. Using radiolabelling, the authors demonstrate that 12 h old IGs are increasingly released upon glucose stimulation and/or NOC treatment. Although Gao mutant islets appear to increase insulin secretion (total or 12 h old IGs), the effects appear to be additive, rather than related to the MT-mediated effects of NOC.*

*In general, data representation must be improved, for example using dot plots rather than bar graphs, especially where only low replicates are available. In these situations, non-parametric testing is superior to parametric testing (for example Mann-Whitney vs t-test).*

*The final paragraphs should speculate a bit more about the role of Gao to put it into context here, as the final result suggests no connection to MTs.*

**Response:** We thank this reviewer for summarizing the data here. We have now improved the data presentation as suggested (paying particular attention to statistical analysis). We have also added more discussion on how Galphao may work.

**Minor**

***Critique 1*** - It would be preferable to replace the term “IGs” to “insulin SGs”, since the latter more commonly used.

**Response:** Done as suggested.

***Critique 2*** *- the term "docked" SGs should be avoided whenever the analyzed images are obtained from fixed samples. In this instance one cannot indeed determine the residence time at the PM of the organelles, which may just be transient visitors. Moreover, “docking” implies a proximity <20 nm, i.e. below the resolution of fluorescence microscopy.*

**Response:** The word (docked) is no longer used in the manuscript. We used “SGs at/close to PM” as alternative.
