# Supplementary material for: Microtubules and Gαo-signaling modulate the preferential secretion of young insulin secretory granules in islet β cells via independent pathways
Source: PLoS One. 2021 Jul 22;16(7):e0241939. doi: 10.1371/journal.pone.0241939 (PMC8297875; doi:10.1371/journal.pone.0241939)
Supplement: Attachment — Submitted filename: Galphao_age Rebuttal 2.docx [file pone.0241939.s005.docx]

We thank both reviewers for their constructive suggestions to improve and further clarify the message in the paper. Below are our responses to all the new critiques.

**Reviewer #1:**

This paper dealing with insulin granule dynamics is a follow-up study from Du’s group of a recent paper on microtubule (ref 34) and an older study on Galphao (ref 15) and as such part the data partially recapitulate previous observations. The authors show here by using nocodazole treatment of mouse pancreatic islets or islets recovered from Galphao mouse that the intracellular distribution of insulin granules is modified. Following these observations, they further blocked protein synthesis with cycloheximide and conclude that older insulin granules are preferentially secreted when microtubules are disrupted.
In this revised version the authors have adequatly addressed all my concerns.

**Response:** We thank this reviewer for the positive opinion.

**Reviewer #2:**

**Overall comments:** Hu and colleagues have made a considerable effort to address our original comments. Accordingly, the manuscript is now much clearer and significantly improved. There are however some aspects of it, which remain to be clarified.

**Response:** We thank this reviewer for the positive comments. The specific comments are now addressed one-by-one as below.

**Specific comment 1:** Figure 1: The authors changed the quantification of insulin levels from confocal images according to our suggestions. However, now one can see that upon nocodazole treatment insulin levels are strongly reduced, while the percentage of insulin in the periphery is higher. What is the interpretation of these results?

**Response:** The exact mechanism for this observation is not clear. We have provided potential explanations in the re-revised manuscript (lines 200-203). “…a parsimonious explanation is that insulin SGs can move to near the PM in the absence of MTs, where SG – PM associations (e.g., via SNARE complex formation) or other unknown mechanisms can trap these SGs near the PM, which facilitates their stimulated secretion”

**Specific comment 2:** Figure 2: How many times have these experiments been independently repeated? This figure could be visually more appealing and easier to interpret if the plasma membrane would be marked, for example with arrows or a coloured outline.

**Response:** Two independent studies were done, with each embedded, sectioned, and imaged separately. “Double-blind tests” were used in the sense that the images were captured by personnel in the Vanderbilt TEM core without knowing what parameters will be quantified. We have now marked the beta-cell membrane with red-dashed lines as suggested.

**Specific comment 3:** Figure 3: The radiolabeling measurements provide information only about the secretion of the young secretory granules, meaning that conclusions about the behaviour of the old secretory granules remain speculative.

**Response:** We agree that we have no direct evidence about the behaviors of old vesicle. The conclusion that disrupting MTs could mobilize older granules was based on the findings that NOC-treated islets secret: (1) smaller portion of young insulin (Figure 3G) but (2) bigger portion of their total insulin (data now added as re-revised Figure 3H). These findings suggest that NOC-treated islets have secreted a bigger portion of older SGs. This line of reasoning has been added to the re-revised manuscript (lines 303-307).

Notably, cycloheximide treatment did not significantly affect insulin secretion. A limitation of this approach, which would need to be acknowledged, is that each stimulation induces only the secretion of a small fraction of the granule stores. Thus, one would need to repeat the cycloheximide treatment multiple times (which is however incompatible with cell viability) in order to reliably assess the contribution of insulin biosynthesis to secretion.

**Response:** We agree and have now acknowledged this limitation in the re-revised manuscript (lines 292-301), which is also a reason why the radiolabeling studies are necessary to support our overall conclusion.

**Specific comment 4:** Figure 4: As in figure 3, the claims about the old secretory granules are speculative. As we requested, the graphs now show the single values, and it seems there are many extreme values that make the comparison significant.

**Response:** For the first part of the critique, please see response to point #3 above [note that Galphao mutant islets secrete less young insulin (Figure 4F), but more total insulin (Figure 4G, middle panel and data in Zhao et al., 2010).

For second part of the critique, all the secretion assays (Figure 4F-G) were done in at least two different days, likely accounting some differences in radioactive aa incorporation (Figure 4F). In addition, the basal insulin secretion in mature islets are usually low (Figure 4G, the left panel), approaching the lower detection limits of most ELISA kits and can produce some outlier reading. In fact, if we remove a “single value” that appears extreme in any sample-group, all comparisons related to that group will yield better p-values in supporting our conclusion. Thus, we included all we had obtained and performed statistical analyses that are suitable for different sample groups (according to if multiple comparisons will be used and how big is the “n”).

**Specific comment 5:** Line 195: "... movement of SGs to endosomes for degradation." – the authors should probably refer to “lysosomes” rather than to “endosomes”

**Response:** changed to “lysosome”.
